# Basic description and some notes on the evolution of seven sympatric morphs of Dolly Varden *Salvelinus malma* from the Lake Kronotskoe Basin

**DOI:** 10.1002/ece3.3806

**Published:** 2018-02-04

**Authors:** Grigorii Markevich, Evgeny Esin, Liudmila Anisimova

**Affiliations:** ^1^ Kronotsky Nature Reserve Yelizovo Russia

**Keywords:** “charr problem”, diversification isolation, functional morphology, spawning, trophic polymorphism

## Abstract

The study examines the basic morphological and ecological features of Dolly Varden from Lake Kronotskoe (Russia, Kamchatka). Seven valid morphs different in head proportions, feeding, timing, and place of spawning have been determined in this ecosystem. The basic morphometric characteristics clearly separate Lake Kronotskoe morphs from each other, as well as from its potential ancestor (Dolly Varden). According to CVA analysis, the most notable morphological characteristics determining the mouth position are the length of a lower jaw and rostrum. Furthermore, five of seven morphs inhabit different depth zones of the lake and feed on different food resources. Our data suggest that reproductive isolation may be maintained by temporal/spatial isolation for two morphs with lacustrine spawning, and by spatial isolation only for the rest of the morphs with riverine spawning. The sympatric diversity of the Lake Kronotskoe charrs is exceptionally wide, and there are no other examples for seven sympatric morphs of genus *Salvelinus* to coexist within a single ecosystem. This study puts forward a three‐step hypothetical model of charr divergence in Lake Kronotskoe as a potential ground for future studies.

## 1 INTRODUCTION

The long‐standing issue of sympatric diversification in the lakes of high latitudes inevitably addresses us to the compelling “*charr problem*.” Charrs are distributed all over the north of Holarctic and are common species for the freshwater ecosystems in the Arctic, the north Atlantic, and the north Pacific basins. These fishes are considered to exhibit exceptional cases of sympatric polymorphism within the range (Behnke, 1972, 1980; Gordeeva, Alekseyev, Matveev, & Samusenok, 2014; Wilson et al., 2004). Numerous groups of charrs demonstrate an evident distance in body and especially head shape in sympatry (Jonsson & Jonsson, 2001). However, despite strong evidence on morphological diversification and clear distinctions in lifestyle among morphs, ecological mechanisms of diversification process are still not clear.

The diversification is usually closely tied to trophic adaptations that implies sharing the food resources. The most commonplace and direct diversification alternation is through diverging along the pelagic‐benthic resource axes (Klemetsen, 2010). This type of evolution trend is the most common for Arctic charr *Salvelinus alpinus* (Linnaeus, 1758) sensu stricto and occurs polytypically more than 40 times across the area of their distribution (Jonsson & Jonsson, 2001; Wilson et al., 2004). The sympatric diversity is usually represented by two or three groups: planktivorous, piscivorous, and benthivorous (Adams et al., 2003; Alekseyev, Pichugin, & Samusenok, 2000; Alekseyev, Samusenok, Matveev, & Pichugin, 2002; Klemetsen, Amundsen, Knudsen, & Hermansen, 1997; Sandlund et al., 1992). Another highly polymorphic charr—Lake trout *Salvelinus namaycush* (Walbaum, 1792)—inhabits large postglacial lakes of North America. Apart from the basic type of speciation, this charr shows some additional pathways leading to the formation of several deepwater or shallow‐water morphs (Chavarie, Howland, & Tonn, 2013; Harris et al., 2015; Muir, Hansen, Bronte, & Krueger, 2015). Both arctic charr and lake trout in a greater or lesser degree are adapted to lacustrine environment, prefer to spawn in lakes, and incline to choose residential life strategy. On the contrast, the mainly anadromous group of charr species hardly ever or even never forms sympatric morphs in the lakes. Reproduction of fishes growing in freshwaters is mostly associated with rivers, while lakes commonly tend to be used as transition waterbodies during migration (Behnke, 1980; Dennert, May‐McNally, Bond, Quinn, & Taylor, 2016; Esin, Bocharova, Mugue, & Markevich, 2017). In particular, no lacustrine morphs were found for *Salvelinus leucomaenis* (Pallas, 1814) and *Salvelinus confluentus* (Suckley, 1859) (Chereshnev, Volobuev, Shestakov, & Frolov, 2002; Dunham et al., 2008). Only two cases of tropic diversification within the lake ecosystem were described for northern Dolly Varden *Salvelinus malma* (Walbaum, 1792); and both of them were observed on the Kamchatka peninsula (Ostberg, Pavlov, & Hauser, 2009; Savvaitova & Kohmenko, 1971; Viktorovsky, 1978).

The phenotypic variability of northern Dolly Varden is mainly realized through various strategies ranging from anadromous to stream resident (Mochnacz, Schroeder, Sawatzky, & Reist, 2010; Pichugin, 2015). The lakes are typically used for growing, yet not for spawning. Only one population from Kamchatkan Lake Azabachye is known to split into benthivorous and piscivorous morphs (Glubokovsky, 1995; Oleinik, Skurikhina, & Brykov, 2010; Savvaitova & Kohmenko, 1971). However, due to volcanic activity in the North Pacific region, numerous lakes were formed by lava dams. Some of them are inhabited by landlocked Dolly Varden that adapted to the lacustrine conditions. Dolly Varden in these lakes is typically represented either by omnivorous slow‐growing populations or exhibits horizontal transformations sensu Adams (1999) into “normal” morph and cannibals. The only complex variant of sympatric divergence in Dolly Varden was found in Lake Kronotskoe to the East of Kamchatka. Different studies report on 3 to 7 morphs in this ecosystem (Markevich, Esin, Saltykova, et al., 2017; Pavlov, Kuzishchin, Gruzdeva, Senchukova, & Pivovarov, 2013; Viktorovsky, 1978). Moreover, they were proved to originate from anadromous Dolly Varden through genetic analyses (Ostberg et al., 2009; Senchukova, Pavlov, Mel'nikova, & Mugue, 2012). Seven distinct morphological types have never been observed previously for any sympatric complex in genus Salvelinus that makes the investigation of morphological and ecosystem features of the Lake Kronotskoe morphs necessary for understanding more complicated mechanisms of diversification.

Meanwhile, little is known about the morphology of these morphs as well as their feeding and spawning biology. As it was mentioned above, Dolly Varden is well‐adapted to anadromy and usually spawns in rivers or brooks. Conserving these features in case of isolation in the Lake Kronotskoe basin may have resulted in specific diversification patterns which were not previously observed both in Arctic charr and Lake trout.

Consequently, the objective for this study was to revise the diversity within the charr morphs from the Lake Kronotskoe basin based on their ecological and morphological features. Outlining the mechanisms of diversification in Lake Kronotskoe may expand the understanding of the “charr problem” in general.

## 2 MATERIAL AND METHODS

### 2.1 Study area

Lake Kronotskoe is situated on the eastern shore of the Kamchatka peninsula in the Kronotsky Nature Reserve. The basin area is 246 km^2^, the average (max) depth is 58 (136) m, the a.s.l. is 372 m, the catchment area covers approximately 2,330 km^2^. The lake has a developed tributary system comprising both small plain‐type brooks with the altitude difference from the head to the mouth approximating to 30–50 m as well as big branched rivers with the drop height of approximately 300–500 m. The ecosystem originated 12–14 thousand years ago due to the volcanic eruption when lava had blocked the valley of the ancestral river thus forming the lake in the upper part of the basin (Braitseva, Melekestsev, Ponomareva, & Sulerzhitsky, 1995). The Kronotskaya River flows down from the lake through the rapids impassable for anadromous fishes (Kurenkov, 1977; Viktorovsky, 1978).

The lake belongs to the dimictic type: the process of water mass mixing occurs in June and at the end of October; the epilimnion reaches 20–25 m at the end of August. Around 80% of zooplankton biomass concentrates in this upper zone. The shores, littoral zone, and small islands are formed by lava boulders from the surrounding volcanoes. The shallow stony areas are characterized by high production of gammaruses, snails, chironomidae, and stone‐fly larvae. The profundal zone is covered with soft grounds; clams, oligochaetes, and chironomidae larvae abound (Kurenkov, 1978).

The fish community is represented by two species: Dolly Varden and kokanee (landlocked *Oncorhynchus nerka* [Walbaum, 1972]). Both species exhibit the trophic‐based diversification and jointly use all the food resources available in the lake (Kurenkov, 1977; Viktorovsky, 1978). All charr morphs were traditionally named with respect to the head shape with the sole exception. The first data about predatory longhead (L), benthivorous nosed (N), and omnivorous white (W) morphs were obtained in 1970s (Viktorovsky, 1978). Lately two deepwater morphs—bigmouth (B) and smallmouth (S) charrs were described from the lake basin (Markevich, Esin, Saltykova, et al., 2017). Recently, it has been identified that the previously described N‐morph is actually represented by three morphotypes which were named N1 (blunt nosed), N2 (sharp nosed), and N3 (shovel nosed)‐morphs, respectively (Markevich, Esin, Busarova, Knudsen, & Anisimova, 2017). Two ephemeris morphs (riverine relic Dolly Varden and Dwarf charr) were also described in the ecosystem (Ostberg et al., 2009; Pavlov et al., 2013). In this study, the first one was reduced to an immature W‐morph (discussed below), while the second one was described on the four specimens only and have never been caught since; therefore, it is doubtful that it may be associated with any of the seven morphs.

### 2.2 Field sampling

The material was collected during the ice‐free period lasting from June to October 2013 and 2014 ubiquitously across the lake basin. Fish was caught in the 60–90‐m lines of multipanel gill nets 20–50 mm mesh size at the depth from 2 to 100 m. The estimation of fish abundance was based on the results of fishing in all relevant habitats: the lower reaches of the tributaries, littoral (depth < 10 m), sublittoral (10–20 m), epilimnion (<20 m), hypolimnion (>20 m), and profundal (>40 m) zones. No less than 20 gill net samplings were performed per zone in various parts of the lake including different bays, areas around islands, banks, and river mouth vicinities (Figure S1). To get a comprehensive picture, we tried to invest the same fishing efforts in different parts of the lake according to the depth profiles, winds, temperature conditions, etc. (see Figure S1). The fishing operations were performed simultaneously in all zones once in 2 weeks, that is, gill nets were exposed in littoral+sublittoral, epilimnion, hypolimnion, and profundal zones roughly at the same time oat each station according to the scheme represented in Figure S1 for the sites near the shoreline and the banks in the open waters. The exposure time varied as following: in littoral and sublittoral zone it lasted for 3–6 hr, in epilimnion and hypolimnion—12–15 hr, in profundal—6–8 hr. Totally 1,232 specimens were caught. Additionally, 27 prespawners of anadromous Dolly Varden from the nearest river basin (the Komarova River) were sampled in September 2013. Sampling in the Komarova River was carried out by gill nets with 20–55 mm mesh size. All fishes were caught in the lower course of the river before they reached the reproduction sites.

### 2.3 Primary separation of morphs

**Figure 1:**
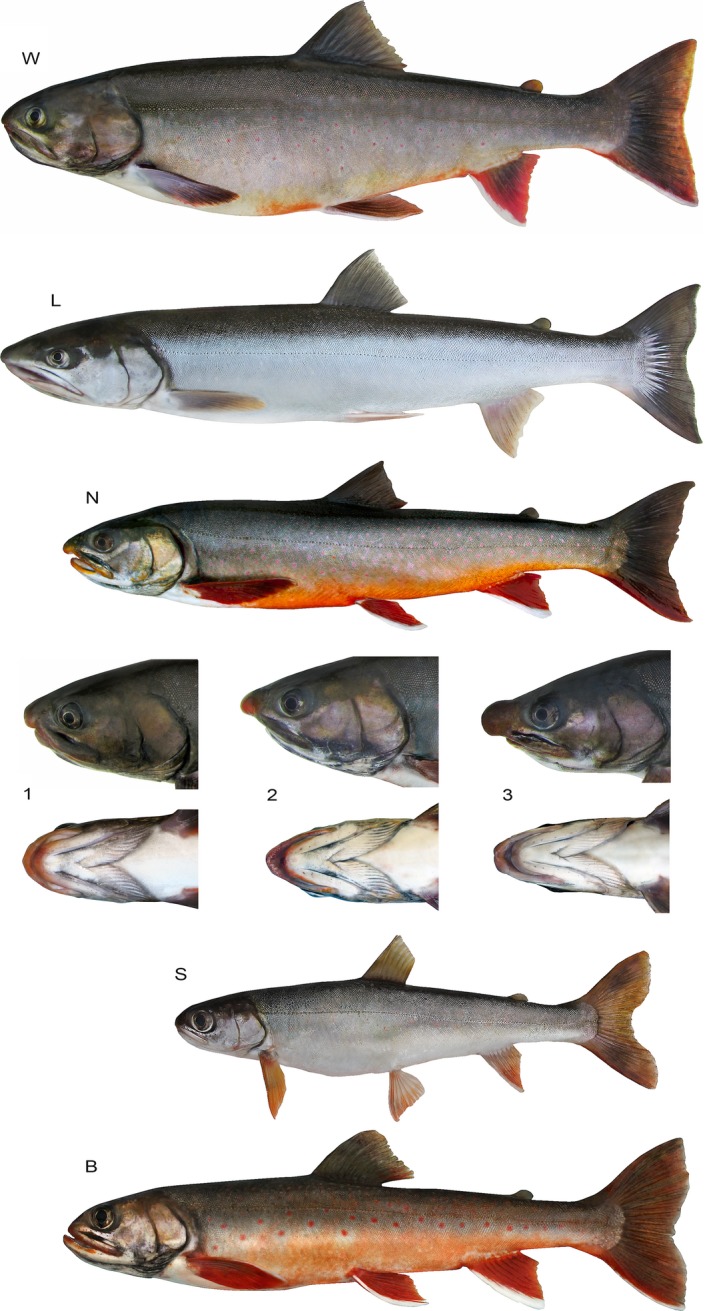
Sympatric morphs of Lake Kronotskoe charrs. W, white; L, longhead; N, nosed: 1—blunt nosed, 2—sharpnosed, 3—shovelnosed; S, smallmouth; B, bigmouth

The diversity of morphs was initially estimated during the catch checking. All charrs were separated into seven groups according to their head length, mouth position, and coloration (Table 1, Figure 1). N‐morphs were further additionally differentiated by the shape of the head. The first group (N1) had the oval shape of the snout, while N2‐morph had a sharped rostrum and N3‐morph—hypertrophied flat rostrum. Furthermore, N2‐ and N3‐morphs unlike N1‐morph were characterized by an uncovered palate (shortened lower jaw), both jaws were covered by connective tissue (“lips”). N3‐morph featured the well‐developed connective tissue plates that covered the snout and formed a sharpened spade‐like rostrum. In N2‐morph, the connective tissue plates turned out to be absent. No difficulties were experienced during the primary separation of morphs. All specimens were sorted by groups unambiguously, and no morphological continuum of variation was revealed.

**Table 1:** Differentiation of morphs in mixed gillnet catches

Symptom	Morphs of charrs [number of individuals]				
	White (W) [346]	Longhead (L) [80]	Nosed (N1‐3) [243/78/42^a^]	Smallmouth (S) [209]	Bigmouth (B) [233]
Head shape	Medium length, conic	Very long and elongated	Medium length and height	Short, conic	Medium length and height
Mouth position	Terminal	Terminal	Bottom	Terminal	Top
Maxilla size and shape	Medium long, slightly curved	Very long and arcuate	Medium long, curved	Short, straight and slim	Medium long and straight
Flanks coloration	Pale, pelagic	Pale, pelagic	Dark	Pale	Dark
Special features	No	Torpedo low body	Overhanging snout	Wedge‐shaped protrusion on belly	Arch‐like lower jaw

a Total quantity of N1/N2/N3 charrs.

### 2.4 Morphology analysis

Geometric morphometric methods were applied to analyze the morphological differences between the groups of charrs. Totally, 322 fish samples were randomly subsampled from the Lake Kronotskoe catches (approximately 20% for abundant morphs and 50% for rare ones). All 27 fishes from the Komarova River, which is southward to the Kronotskaya River basin, were analyzed. The left side of these specimens was photographed by Canon EOS 350D with 50 mm lens in the orthogonal projection, fins straightened, and mouth closed. In addition, the bottom view of the head was photographed for N‐morphs. To circumvent any optical distortions, the longer side of the image was always set to fit at least twice the fish length, and the same values of the focal length (80 mm) were used in imaging. Totally 23 landmarks were digitized for each fish (Figure S2) using a tpsUtil and tpsDig2 v2.16 software platforms (Rohlf, 2010).

The comparison was performed in three steps. First, the differences among the Lake Kronotskoe morphs were normalized against the anadromous Dolly Varden. Then the differences among five main morphs (all nosed charrs combined), and among three N‐morphs were evaluated. The analysis was carried out by MorphoJ 1.06d (Klingenberg, 2008). A generalized Procrustes superimposition (Dryden & Mardia, 1998) was applied to minimize the distances between individual landmark configurations (Zelditch, Swiderski, Sheets, & Fink, 2004). Therefore, the Procrustes distances became relative measurements of the shape differences among the groups, and a general Procrustes dispersion analysis was used to estimate the divergence among the groups (Rohlf & Slice, 1990). Sexual dimorphism was not profound in the measured traits (Procrustes ANOVA *F*_318,13558_ = 12.7, *p *=* *.083); thereafter, all the calculations were performed on both sexes combined. To cross‐validate our preliminary classification Jack‐knife was used for an unbiased estimation of the morphs' classification.

Canonical variates analysis (CVA) was applied to assess the total amount of variation in body shape among the groups. This method reduced the amount of variation within the groups and made the variation among them more vivid (Klingenberg, Barluenga, & Meyer, 2003; Zelditch et al., 2004). Multi‐dimensional plots were used both to describe the morphospace (i.e., the abstract space where each point represents a particular individual) and to identify the differences between the groups. To evaluate the contribution of dispersion of individual landmarks to the General discrimination of the groups' body shape, canonical loadings of landmarks on the first three axes were estimated. To visualize the shape changes in the groups, the average head and body shapes for the sets of individuals were compared with the consensus shape of all fish subjected to analysis.

### 2.5 Fish size differentiation

Fork length (FL, mm) and weight (*W*, g) were measured for all specimens, and multivariate dissimilarities in fish size were statistically examined using post hoc ANOVA Tukey's HSD. The mature status was determined according to Eenennaam and Doroshov (1998) recommendations, wherein all specimens with gonad development over III stage were considered as mature (1), fishes with the gonad development lower than III stage—as immature (0). Then, each set of fish was sorted by the fork length, and the mature statuses were plotted as a function of length. The mature status plots were averaged using 10‐point running average procedure, and approximated by a simple exponential function:

$$M=1-e^{\frac{{\text{FL}}_{0}-\text{FL}}{{\text{FL}}_{e}}}$$

where *M* is the average mature status, FL_0_ is the minimal length of maturation, and FL_*e*_ is the characteristic length of maturation. Thus, the average 50% maturation fork length was calculated as:

$${\text{FL}}_{50\%}={\text{FL}}_{0}-{\text{FL}}_{e}\times \log\;1/2$$

### 2.6 Analysis of fish biology

The analysis of stomach content was carried out in the field laboratory within 3‐5 hr after the fish sampling. Food objects were identified according to Manko (2016) recommendations by means of stereomicroscope MBS‐9 (12‐20x). Prey from the stomachs of all the Lake Kronotskoe charrs was sorted into the following groups: chironomidae larvae, chironomidae pupae, stone‐fly larvae, insect imagoes, gammaridae (*Gammarus* spp.), oligochaetes, snails (*Anisus* spp. and *Limnea* spp.), clams (*Pisidium* sp.), fish. The occurrence of these food objects was estimated for charr groups as

$$\text{FO}=\frac{N_{i}}{N_{\mathrm{tot}}},$$

where *N*_*i*_ is the fish number for each morph with the specified prey in the stomach, *N*_tot_ is total fish number for each morph with the stomach content (Amundsen, Gabler, & Staldvik, 1996).

The observation of the spawning grounds was carried out within the period from August to October of 2011–2015 in the basins of main tributaries (4 rivers and 5 brooks) and till December in the lake. Additionally, the lake area was studied in March–April 2011 to define the B‐morph spawning period.

The first step of the observation aimed to define the beginning of the spawning migration in different rivers and brooks. Every 2 weeks, the lower courses (1–2 km) of each river/brook were visually inspected, the prespawning fish was defined by the development of spawning coloration, thus the beginning of spawning migration was determined. The second step focused on determining the reproduction sites and confirming the stability of spawning distribution. All rivers chosen for our analysis were totally inspected via rafting or hiking from the riverheads to the rivermouths no less than 2 times in separate (discrete/different) years (Figure S1). The third step comprised the regular observations at several spawning sites of all morphs from the beginning to the end of spawning period in order to define the reproduction period and confirm the spawning isolation between the existent morphs (Figure S1).

Fishing of spawners at all three steps was implemented according to the catch and release principle by fishing rods. Therefore, spawners were not used for any analysis which included fish killing. Spawning sections in the tributaries were determined by visual observations, the numbers of redds occurrence and females on late IV or V maturation stages were presented at the current sites. The identity of morphs was determined by the aforementioned criteria, and all fish were also photographed to get the additional possibility for morph specifying and to be stored in the personal archive. The description of riverine spawning sites included the mean channel gradient calculated as a ratio of the altitude difference to the river section length in m/km. The distribution over the lake area and the depth range was specified for the lacustrine spawning areas. As it was impossible to visually observe the redds being constructed in the profundal zone, the spawning areas as well as the timeframe were determined by the gill net catches. Only fishes sampled on the V maturation stage were counted as spawners.

## 3 RESULTS

### 3.1 Morphological differentiation of morphs

**Figure 2:**
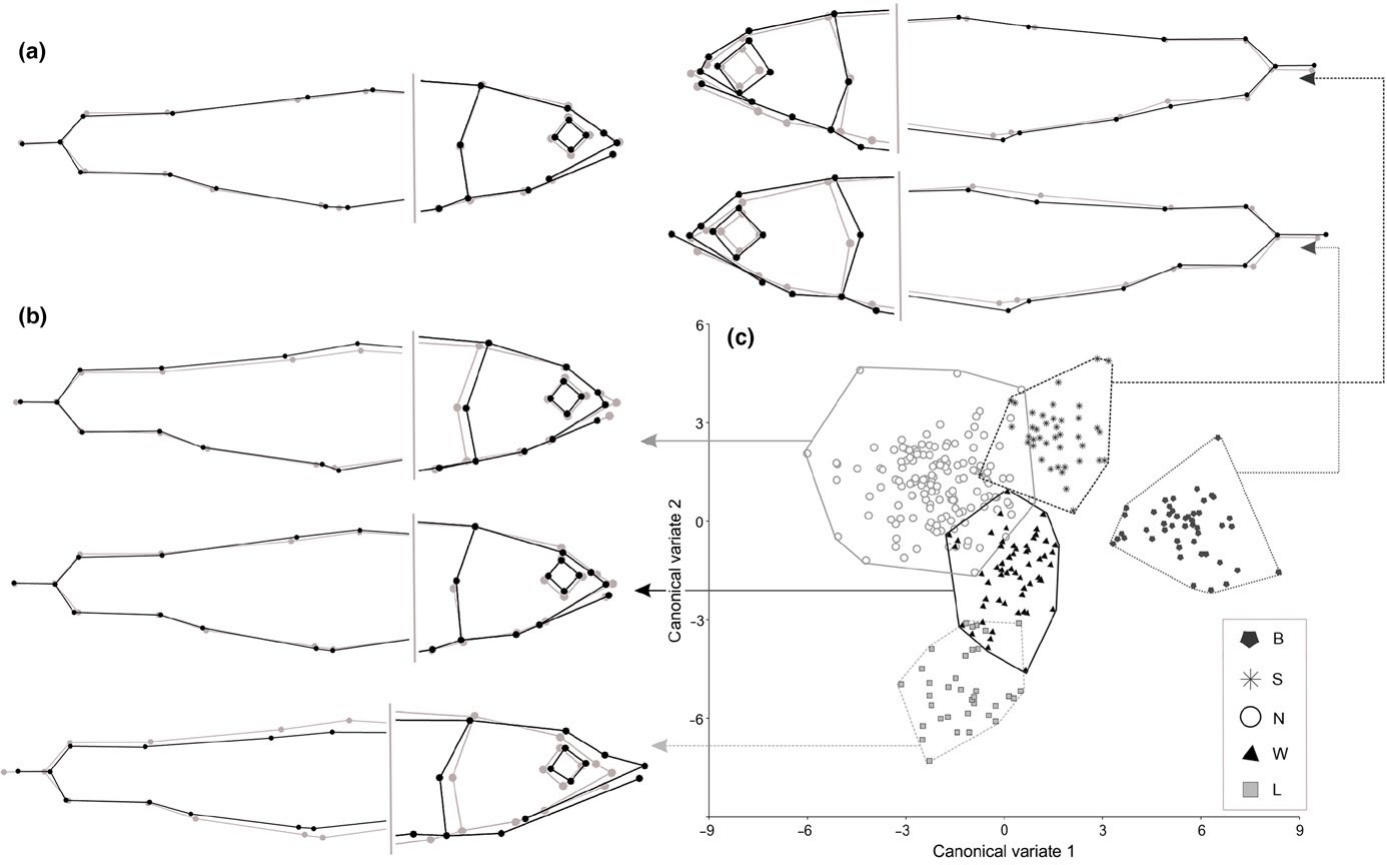
Results of charrs morphological comparison derived from CVA, (a) The averaged Dolly Varden morphological shape against Lake Kronotskoe charrs consensus shape; (b) The shape changes of five main Lake Kronotskoe morphs in relation to the consensus body shape; (c) Plot of specimens of five main Lake Kronotskoe morphs in CV1–CV2 morphospace

The first step of morphological comparison was to distinguish the specific features in shape for the Lake Kronotskoe charrs as contrasted to anadromous Dolly Varden, presumably being ancestral for all the lake morphs. The results of Procrustes dispersion analysis indicated each endemic sympatric morph to be different from the riverine Dolly Varden in a set of morphological features (*F*_318,1356_ = 8.36, *p* = .003). In general, the exterior proportions of Dolly Varden turned out to be very close to the consensus morphological shape derived from CVA of all the Lake Kronotskoe charrs combined (Figure 2a). No traits of head morphology were found to distinguish Dolly Varden from W‐morph; only fins shifted to the head placed apart the anadromous charrs in this comparison. In this regard, a posteriori Jack‐knife cross‐validation had moderate success in Dolly Varden classification against W‐morph, only 52% of the anadromous charrs was classified correctly (Table S1). Herewith, N‐morphs had comparatively shorter jaws and a higher nape. S‐ and B‐morphs differed predominantly in eye diameter and jaw length. L‐morph was distant from the Dolly Varden in a much lower head, longer snout, gill cover, and jaws. The differences described above can be recognized quite clearly based on linear measurements of charr heads (Table S2).

The next step of comparison indicated that the body shape is different among the five main Lake Kronotskoe morphs (Procrustes ANOVA *F*_294,1375_ = 12.6, *p *<* *.001). The most distant groups were deepwater B‐morph and epilimnetic L‐morph, while W‐morph took the closest position to the center of the grid. N‐morphs turned out to be closer to W‐morph; hypolimnetic S‐morph was closer to the second deepwater B‐morph (Table 2). The maximal internal morphological diversity characterized N‐morph, whereas L‐ and B‐morphs were minimally polymorphic.

**Table 2:** Procrustes distances among morphs (and their variation within the group, %)

Morph (var.)	Morphs			
	L (11.893)	W (22.859)	N (29.067)	S (18.432)
W (22.859)	0.168	0	0.159	0.189
N (29.067)	0.374	0.159	0	0.164
S (18.432)	0.387	0.189	0.164	0
B (17.749)	0.296	0.218	0.292	0.206

	Nosed charrs morphs			
	Side view		Head ventral view	
	N1 (38.352)	N2 (31.253)	N1 (37.313)	N2 (33.564)
N2 (31.253/33.563)	0.010	0	0.086	0
N3 (30.395/29.124)	0.015	0.012	0.063	0.092

CVA of the landmark positions resulted in outlining seven significant CVs differentiating the morphs (Table S3, Wilk's lambda < 0.001). The degree of divergence between the morphs is illustrated in CV1–CV2 space (Figure 2c). According to the canonical coefficients (Table S4), the first CV root (41.3% of total variation) primarily separated sets of specimens in the position and length of the lower jaw, eye diameter, and width of the caudal peduncle. This root differentiated both deepwater B‐ and S‐morphs from the others mainly due to a big eye as well as the two deepwater morphs from each other due to a jaw length. The second CV root (29.1% of total variation) was associated with the dissimilarities in the shape of the front part of the head and jaw position. L‐, N‐, and W‐morphs were clearly distinct from each other along the second root wherein elongated‐head morph with big mouth (L‐) and conical‐head morph with short jaws (N‐) occupied the opposite poles. W‐morph was characterized by mean values of morphological traits (Figure 2b). Jack‐knife comparisons indicated that on the average 92% of charrs were classified correctly (Table S1) with minimal classification probability for W‐morph (84%) and maximal for B‐morph (100%).

Clear morphological differences across three N‐morphs were also revealed. The use of Procrustes dispersion analysis demonstrated the differentiation among the groups to be evident either in side‐view head and body shape (*F*_88,1620_ = 2.01, *p *<* *.010), or in ventral‐view head shape (*F*_32,1760_ = 11.02, *p *<* *.001). Along with it, the relative Procrustes distances between the groups were smaller in comparison with the distances across all five main morphs (Table 2). The maximal internal morphological diversity characterized N1‐morph while N3‐morph was minimally polymorphic and more distant from the others.

**Figure 3:**
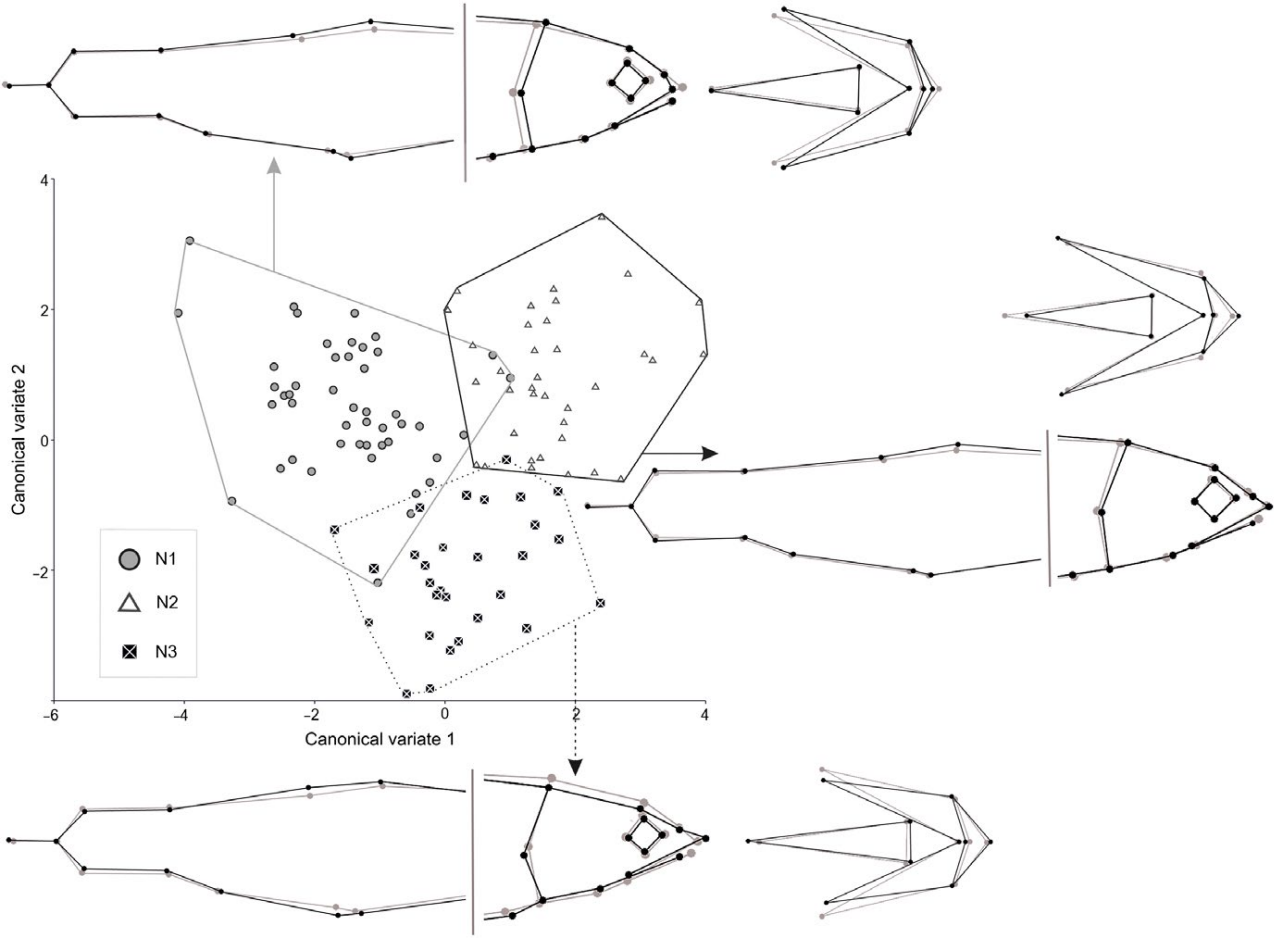
Results of N‐morphs morphological comparison derived from CVA. Plot of specimens of three morphs in CV1–CV2 morphospace surrounded with the associated shape changes in relation to the consensus body shape of N‐charrs

Variation in head and body shape of N‐morphs was associated with three CV roots (Table S3, Wilk's lambda < 0.001). Based on the canonical coefficients of side‐view CVA (Table S4), the first root (72.2% of total variation) primarily differentiated the morphs in the lower jaw length and the caudal peduncle height. The second root (22.5% of variation) mainly separated the morphs in the eye diameter and the height of the caudal fin base. CVA of ventral‐view head shape separated the groups in the rostral length and the width of palate bare part along the first root (52.1% of total variation), while the second root (42.2%) was associated with the head width. The groups had the minimal transgression in CV1–CV2 morphospace (Figure 3). As compared to the consensus shape, N1‐morph had a shorter snout and an upper jaw; N2‐morph had a bigger eye, the maximal bottom‐view length of the rostral part and a narrower distance between the nostrils; N3 had a narrower snout, the minimal eye diameter and length of lower jaw as well as the increased bottom‐view back angular length. Jack‐knife comparisons indicated high classification success for N‐morphs (Table S1).

Thus, all the groups initially classified by the qualitative characteristics during the gillnetting significantly differed by the head and body morphology. This outcome of our comparison confirms the existence of seven morphs in the ecosystem of Lake Kronotskoe.

### 3.2 Size and weight differences

Descriptive characteristics of the seven morphs are represented in Table 3. The maximal body size was observed for W‐ and L‐morphs, while three N‐morphs were smaller and close to each other in their maximal length and weight. Lacustrine morphs (B‐ and S‐) had the smallest mean body size. The average length of adult fish was maximal for L‐morph. Herewith, the maximal length at 50% maturation was observed for W‐morph. Among N‐morphs, both characteristics were the highest for N2‐morph. B‐ and S‐morphs were characterized by the lowest value of both traits. The difference in fork length for mature fish was examined using ANOVA (*F*_6,319_ = 52.2). Thus, S‐, B‐ and L‐morphs were significantly differentiated from each other as well as the other morphs (Tukey's HSD *p = *.00003–.00007). No considerable difference was found in body length between three N‐morphs and W‐morph (0.207–0.751).

**Table 3:** Size‐weight characteristics of charr morphs

Morphs (spec.)	FL_max_, mm	*W*_max_, g	FL_mean_ (mature), mm	FL (50% maturation), mm
W (323)	710	2,870	370 ± 6.5	363
L (71)	657	2,550	491 ± 13.9	347
N1 (277)	413	640	326 ± 2.7	310
N2 (79)	420	660	352 ± 5.7	346
N3 (55)	462	700	336 ± 15.3	254
B (222)	358	360	274 ± 1.7	227
S (205)	330	143	204 ± 1.4	158

### 3.3 Distribution patterns and feeding

Our investigation revealed that the assigned morphs are characterized by the set of specific features of distribution in the lake ecosystem (Table S5). Two of the morphs predominantly inhabit the deep water zone accounting for more than 50% of the gillnet catches on the depth exceeding 20 m. Herewith, B‐morph is dominant on soft grounds near the bottom (>40 m), while S‐morph can be mainly found in hypolimnion water column (>20 m). More than 50% fish in littoral (2‐20 m) gillnet catches were N‐charrs, and no microspatial differences were found in the distribution of three N‐morph groups. They all occupy the same sites of stony grounds in the shallows but the first group occurred in the catches more frequently. The epilimnion pelagic area is the only place where L‐morph was abundant (14% of catches in this zone), just sole specimens of L‐morph were found in the other zones. This morph concentrates at the depth of 10‐15 m and can be found all over the lake epilimnion zone. The most abundant W‐morph exhibits no significant spatial preferences and can be found all over the lake since more than 30% of individuals in each zone belonged to W‐morph. The immature specimens were the most numerous in the littoral zone and in the tributary mouths, while adults preferably inhabited epilimnion (75% of fish in gillnet catches), but also occurred in the shallow waters, hypolimnion, or profundal zone.

The dietary specialization of Lake Kronotskoe charrs assessed as the occurrence of prey in the stomachs clearly corresponds to the distribution patterns of the morphs. The most common prey in B‐morph stomachs was chironomidae larvae, oligochaetes, and clams (*FO* =  0.93/0.43/0.48 correspondingly). Hypolimnion S‐morph had a similar, but a wider dietary range. Chironomidae pupae and insect imagoes were found in the stomachs with equal frequency (0.36) alongside with the above‐mentioned organisms (0.88/0.24/0.31 correspondingly). The shallow water N‐charrs mostly consume gammaruses (0.74). In general, no dietary differences were found between three N‐morphs. With the exception of about 25%, N1‐morph charrs had an alternative feeding strategy of consuming mostly chironomidae larvae and pupae (0.63/0.37), but not gammaruses (0.06). The epilimnetic L‐morph is a typical predator; the stomachs usually contain carcasses of kokanee, yet rarely charrs (0.87). No dietary shifts were found for the above‐mentioned morphs during the growing period in lacustrine environment, while W‐morph clearly exhibits such shift upon reaching the length of 30–35 cm. Small immature fish usually consume benthic organisms (snails and chironomidae (0.67/0.82), but not fish (0.04). Adult fish switch to predatory feeding and majorly consume deepwater charrs and kokanee (0.79), while the occurrence of shallow water invertebrates significantly decreases (0.15).

### 3.4 Spawning

**Figure 4:**
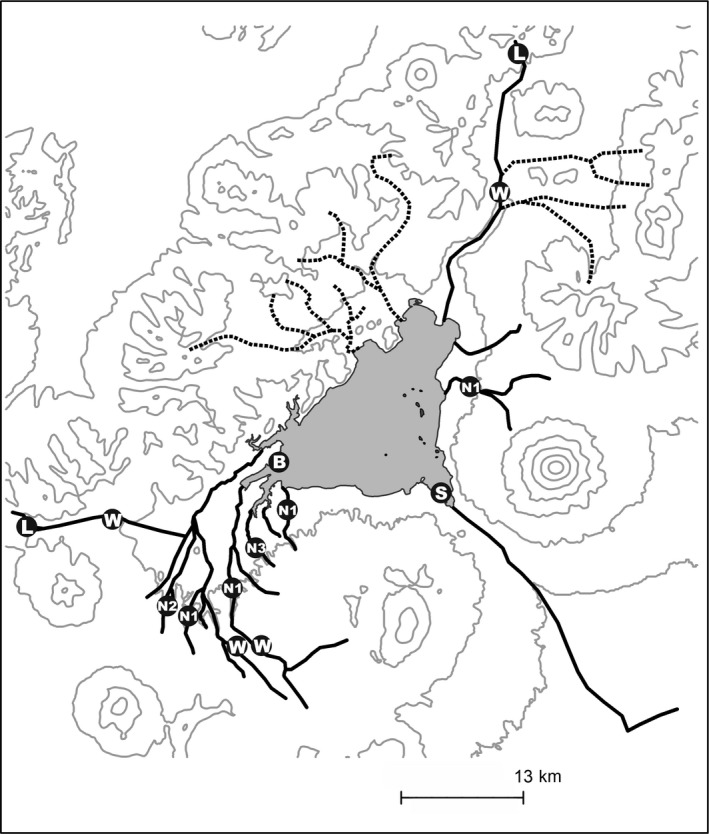
Spawning sites of charrs in Lake Kronotskoe basin (marked with letters for different morphs)

The spawning areas of all seven morphs were documented all over the basin by mapping the reproduction sites either in the tributaries or in the lake. All morphs can be divided into two groups: lacustrine and riverine spawners. Both profundal and hypolimnion dwelling morphs spawn directly in the profundal zone. Herewith, the temporal and spatial division is observed for them. S‐morph spawns from the end of October till the middle of December in the bay near the riverhead of the Kronotskaya River at the approximate depth of 20–40 m (Figure 4). According to the gill net catches, the spawning proceeds in big groups. The spawning specimens had no injuries on the body or the fins. This could indirectly indicate that S‐morph charrs do not construct redds and cast eggs in the cracks of lava stones. The spawning biology of B‐morph is not profoundly studied, but prespawning fish (males with gonads at V developmental stage, females at late IV) was caught only in the south‐east bays of the lake in December at the approximate depth of 50–60 m. The lack of spawning in December and March–April indicates that spawning takes place in January–February.

The rest of the morphs spawn in the lake tributaries from the middle of August till the middle of October. The spawning migration from the lake was defined to begin in the first 10 days of August and end in the last days of the same month. In this period maturating (gonads on the IV stage for males and females, as well as the developing spawning coloration) fish of all morphs was observed in the low reaches (2–5 km) of all rivers and brooks under scrutiny. No maturating fish was found in the low reaches of the river after the beginning of September. During the middle of August—the middle of October the spawning sections were mapped as follows: the sections with compact redds agglomerations (>10 redds) and fish pairs (males on V gonads developmental stage; females on late IV or V) were traced as spawning sites. Clear spatial segregation was found for all riverine morphs either within or among the tributary basins. All N‐morphs spawn in the small brooks with the plain channels or in the middle course of the big rivers with the channel gradient of about 0.1 m/km. Herewith, three N‐morphs demonstrate a clear reproductive confinement to different tributaries. The most abundant N1‐morph spawns in a number of rivers and brooks while N2‐ and N3‐morphs are defined by a narrow spawning distribution. According to the visual observations and catches, the first spawners (both males and females at V gonad developmental stage) appear at the reproduction sites at the end of August; but at the beginning of October no spawning fish was observed at the sites. N2‐morph reproduces in the sole brook with plenty of groundwater outflows during the middle of August until the first week of September. N3‐morph spawns in the only river with the plain meandering channel in the middle of September—the beginning of October (Figure 4). As far as W‐morph spawning grounds are concerned, they predominantly occupy the upper course of the big rivers at the rapid‐pool sites with the channel gradient of about 0.2 m/km. The spawning period lasts from the middle of September until the middle of October. Similarly to W‐morph, L‐morph spawns in big rivers from the beginning till the end of September; yet, the spawning sites are located only in the headwaters beyond the systems of rapids that probably cannot be passed by the other morphs. The channel gradient here is 0.3‐0.4 m/km. It should be specially noted that in case when the reproduction of two or more morphs coincided in the same basin, the spawning sites were separated from each other by a lengthy river section (>1 km) without any spawning fish. This observation was multiply checked during the observations of 2011–2015 years. Thus, joint spawning of different morphs was not observed within this ecosystem.

## 4 DISCUSSION

The complex analysis comprising both the morphological and the ecological characteristics provided the solid ground for outlining seven distinct charr morphs coexisting in the Lake Kronotskoe ecosystem. The morphological analysis has revealed that six morphs are clearly distant from the anadromous Dolly Varden in head and body morphology; yet, only W‐morph has no reliable head shape features which separate it from the anadromous Dolly Varden. It should be noted that the Dolly Varden body shape complied with the consensus while all the other morphs were distant from the consensus in various ways.

Both deepwater morphs, in comparison with epilimnetic ones, had significantly bigger eyes and a shorter snout. The blunt head shape and the large eye are common morphological features of deepwater charrs which were observed for different lakes across the range. In particular, it was previously described for deepwater benthivorous morphs from lakes Skogsfjordvatn (Scandinavia), Kamkanda (Transbaikalia), Loch Dughaill (Scotland) (Alekseyev et al., 2014; Hooker et al., 2016; Knudsen et al., 2016; Skoglund, Siwertsson, Amundsen, & Knudsen, 2015). However, B‐ and S‐morphs are clearly different from each other in jaw length. The jaws of S‐morph are short proportionally to the snout length; thereby, this morph conserves the terminal mouth position, which is common for the omnivorous Dolly Varden. On the contrary, B‐morph has a very long lower jaw that once being combined with a short snout results in the supra‐terminal mouth orientation.

The most reliable characteristic of the three N‐morphs is the sub‐terminal mouth position that is determined by a short lower jaw and a relatively long snout. The shortening of the lower jaw is a trend seldom registered for benthivorous charrs; however, it is common for ciscos and can be regarded as the most wide‐spread adaptation for benthic feeding in a littoral zone (Bronte, Fleischer, Maistrenko, & Pronin, 1999; Lu & Bernatchez, 1999; Østbye, Næsje, Bernatchez, Sandlund, & Hindar, 2005). Different N‐morphs were clearly distant from each other in the lower jaw length and the head width.

L‐morph has an elongated head accompanied by a proportionally longer jaw. This feature can be regarded as a common adaptation for chaise predatory strategy sensu F. Juanes (Juanes, Buckel, & Scharf, 2002) for fishes in general (Alekseyev et al., 2014; Burress, 2015; Saltykova, Siwertsson, & Knudsen, 2017; Skoglund et al., 2015).

To sum up, all trophic‐related morphological adaptations in Dolly Varden morphs from Lake Kronotskoe are formed by the variability in jaw length, which could be either correlated or non‐correlated with the modifications in the snout length. This variability greatly modifies the head shape and shifts the mouth orientation from terminal to supra‐terminal or sub‐terminal position thus giving rise to the necessary adaptations to the existing ecological conditions. The only reliable difference in body shape occurs in the length and the height of the caudal peduncles which turned out to be higher in all N‐morphs, longer in W‐, and S‐morphs, and shorter in B‐ and L‐morphs.

Both head and body shape features are strongly associated with the foraging strategy of each morph. The lack of specific adaptations in W‐morph compared to the anadromous Dolly Varden corresponds to its broad food niche. Deepwater charrs are defined by clear morphological shifts which are common for all deepwater fishes and were described previously (Alekseyev, 2000; Præbel, Ostbye, Hassve, & Hagenlund, 2015; Skoglund et al., 2015). Therefore, these characteristics can be recognized as a general specialization for foraging in poor light conditions which is realized through pedomorphosis. However, S‐morph conserves the terminal mouth position and occupies the omnivorous niche in hypolimnion. The supra‐terminal mouth position predetermines the deep trophic specialization of B‐morph. This group consumes deepwater benthic organisms from soft sedimentary grounds presumably using the lower jaw as a scoop. All the three N‐morphs dwell in the littoral zone and consume invertebrates from stony grounds. Sub‐terminal mouth position can be recognized as a necessary specialization for consuming prey from big stones. The same type of adaptations was observed among rock‐dwelling cichlids in Lake Malawi (Albertson, 2008). Although the similar type of feeding for the three N‐charr groups impedes the understanding of the function, the head shape differences are prominent among them. N1‐morph with the rounded rostrum and well‐developed connective tissue on the jaws strongly reminds us of the hypertrophied lips of barbs and cichlids (Colombo et al., 2013; Dimmick, Berendzen, & Golubtsov, 2001; Roberts & Khaironizam, 2009). Such type of head shape was previously recognized as a vital adaptation for effective food extracting from narrow holes between the stones (Lukas, Gonzalo, Frederico, & Axel, 2015). According to the deep structural transformations of skull, we previously assumed that N2‐ and N3‐morphs are specialized groups of N‐charr, which consume gammaruses using their teeth‐armed bones as pincers (Markevich, Esin, Busarova, et al., 2017). These head morphology transformations allow N2‐ and N3‐morphs to grab gammaruses more effectively. Being a chasing predator L‐morph, is characterized by a cone‐shaped head armed with very long jaws necessary to catch kokanee in the water column. The transformations in the caudal peduncle length and width also strongly correlate to the feeding strategy reflecting the various swimming abilities of different morphs (Robinson & Parsons, 2002).

The definitive fish length and the length at maturation also correspond to the lifestyle. The smallest size parameters were observed for deepwater morphs while the biggest ones for L‐ and W‐morphs were both associated with predatory feeding.

Feeding biology and trophic‐based adaptations determine the morph distribution in the Lake Kronotskoe ecosystem. The only group that can be found in every zone of the lake is W‐morph, and exactly this one occupies the omnivorous niche. Reproduction characteristics also exhibit the clear distinctions among the aforementioned morphological groups. Profundal and hypolimnion morphs spawn only in the lake and, therefore, are isolated from each other by the place and the time of spawning. In contrast, the epilimnetic morphs reproduce almost at the same time of the year but only in riverine spawning grounds. N‐, W‐, and L‐morphs occupy small brooks/semi‐montane river sections, montane sections, and headwaters correspondingly. This distribution is determined by the river gradient increasing from the river mouth to the headwaters. Therefore, the succeeding river sections correlate directly with the increasing velocity allowing the morphs to pass them. The morphs are arranged in the N‐, W‐, L‐ row correspondingly. Moreover, the aptitude to predatory feeding also increases in the same row, wherein N‐morph is purely benthivorous, W‐morph switches to the piscivorous feeding in adulthood and L‐morph is piscivorous throughout the whole life. N1‐, N2‐, and N3‐morphs occupy the distant tributaries which are far remote from each other.

**Figure 5:**
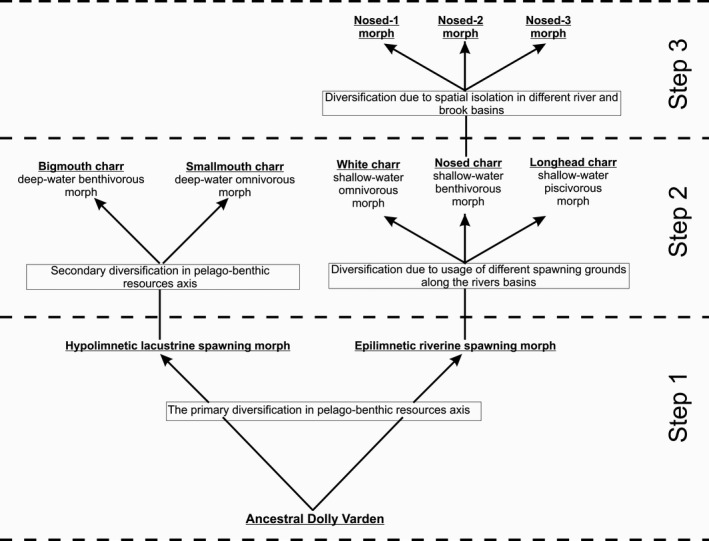
Three steps of Dolly Varden evolution in Lake Kronotskoe

It should be specially noted that common Dolly Varden typically exhibits a high range of phenotypic plasticity which can be further realized in decent morphs through divergent natural selection. The data collected on morphology, ecology, and reproductive biology peculiarities may suggest that a potential divergence model in the Lake Kronotskoe morphs can proceed in three steps (Figure 5).

At the first step after the lake formation the ancestral Dolly Varden could presumably have split into two groups. The first protomorph could have adapted to the lacustrine conditions in the hypolimnetic zone with a narrow diversity of food resources and switched to the lacustrine spawning. The other protomorph could have occupied the epilimnion zone with a wide diversity of food resources and since then maintained the riverine spawning.

At the second step of diversification the deepwater, lacustrine spawning protomorph would have gone through a diversification in the pelago‐benthic resource axes; as a result, two distinct groups, namely B‐ and S‐morphs, may have appeared. Our data demonstrate that they both inhabit the deepwater zone and the isolation is provided by the difference in the place and the time of spawning. At the same step of diversification, the riverine spawning charrs might have split into three groups: L‐, W‐, and N‐morphs. The primary driving factors that lead to the diversification in river‐lake charrs seem to be most logically associated with spawning in different sections of the lake tributaries. The isolation during spawning was associated with different riverbed gradients and determined by the critical current velocity hypothetically passable for each morph.

The third step of the evolutionary model we put forward is currently going on and is manifested in diversification of shallow‐water N‐charrs into three more groups. The isolation during the reproduction period is ensured using the spawning grounds in different remote tributaries of the lake. Although these groups are very similar in feeding biology, they exhibit significant differences in a head shape.

Our morphological and ecological findings support that the polymorphism of Dolly Varden arising in the Lake Kronotskoe can be considered as a highly probable outcome of the multidimensional disruptive natural selection proceeding within a single ecosystem. Notably, the sympatric diversity found in Lake Kronotskoe is exceptional and exceeds any other known cases for genus *Salvelinus* in general. It is well acknowledged that any kind of evolutionary processes in sympatry is based on the balance between the effective feeding and successful reproduction strategies (Schluter, 2001). Sympatric speciation is also impossible without reproductive isolation, which can be realized via spatial, temporal, or behavioral segregation (Bolnick & Fitzpatrick, 2007; Seehausen & Wagner, 2014). The morphs in Lake Kronotskoe exhibit the profound level of trophic‐based specialization in head and body shape which corresponds to their foraging adaptations and distribution within the lake during growth and maturation. Both the spatial and the temporal isolation are comparatively clear during the reproduction period thus implying that the isolation exists among the morphs.

Food chains in Lake Kronotskoe consist of quite typical units that are common for many dimictic lakes in Holarctic (Jónasson, 1992; Whittaker & Fairbanks, 1958). Thus, the diversity of food objects accounting for the variety of food niches cannot be the only factor that promotes the charr diversification along the different pathways in Lake Kronotskoe. At the same time, the riverine spawning and the preadaptation to the riverine conditions is one of the most important features of Dolly Varden biology. The riverine spawning could have shaped the diversification processes and made the morphs more elaborated and diverse.

The charr community in the deepwater zone in its basic features (feeding and spawning) is similar to the numerous cases of Arctic charr lacustrine populations (Adams, Wilson, & Ferguson, 2008; Alekseyev et al., 2002; Jonsson & Jonsson, 2001; Klemetsen et al., 1997). The only important distinction is that in opposition to numerous pelagic morphs observed in sympatric complexes of Arctic charr the S‐morph from Lake Kronotskoe is omnivorous, but not planktivorous which is due to the low abundance of zooplankton in the profundal zone. Moreover, Dolly Varden in general is hardly able to feed on zooplankton due to low‐number and short‐length gill rakers in comparison with Arctic charr.

We examined all the abundant morphs of the Lake Kronotskoe charrs inhabiting all ecological niches available in the ecosystem with the exception of planktivorous specialization. All morphs are distant from each other in morphology, distribution across the lake, feeding and spawning strategies. The evidence on the Lake Kronotskoe charrs shows that the morphs are stable from generation to generation in the given environment. The possible available ecological niches were comprehensively studied allowing us to suggest that the initial description of charr diversity in the basin is complete. Interestingly enough, we have not found “Riverine Dolly Varden” and “Dwarf charr”, which were previously described for this ecosystem (Ostberg et al., 2009; Pavlov, Pivovarov, & Ostberg, 2012; Pavlov et al., 2013). There were no criteria suggested to separate W‐morph and Riverine Dolly Varden in those issues except for the habitat in the river mouths. Consequently, it can be identified as W‐morph that feeds in river mouths or migrates upstream following kokanee for fattening during the spawning period. The second morph was described from the head of the Kronotskaya River, but the description was performed on four individuals only. Thereby, to associate Dwarf‐morph with any of the seven morphs seems to be rather questionable. No more fish with the corresponding characteristics of the Dwarf charr were caught in that area, spawning in the river source was assumed for the Dwarf morph (Pavlov et al., 2012). The Dwarf charr has recently been found to resemble S‐morph by the body shape, but is distinct from the latter by the habitat it dwells in (Markevich, Esin, Saltykova, et al., 2017).

The analysis of mitochondrial DNA polymorphism previously showed a low level of differences between W‐, L‐, and N‐morphs (Ostberg et al., 2009; Radchenko, Salmenkova, & Omel'chenko, 2006; Salmenkova et al., 2005; Senchukova et al., 2012). In this case, the confirmation of reproductive isolation could be analyzed thought the polymorphism of the microsatellite loci and the disruptive selection efficiency through the histo‐compatibility complex. However, it is beyond the scope of the present study and will be discussed in the succeeding papers. Furthermore, it should be noted that the recently discovered morphs (B, S, N2 and N3) have never been analyzed by the molecular methods.

## CONFLICT OF INTEREST

None declared.
