# Supplementary material for: Basic description and some notes on the evolution of seven sympatric morphs of Dolly Varden Salvelinus malma from the Lake Kronotskoe Basin
Source: Ecol Evol. 2018 Feb 4;8(5):2554–67. doi: 10.1002/ece3.3806 (PMC5838070; doi:10.1002/ece3.3806)
Supplement: Supplementary file 1 [file ECE3-8-2554-s001.doc]

**Supplement materials**

**for *Markevich G.N, Esin E.V., Anisimova L.A. Basic description and some notes on the evolution of seven sympatric morphs of Dolly Varden Salvelinus malma from the Lake Kronotskoe Basin***

**Figure S1.** The scheme of gillnet exposition, and spawning sites studies in the Lake Kronotskoe basin


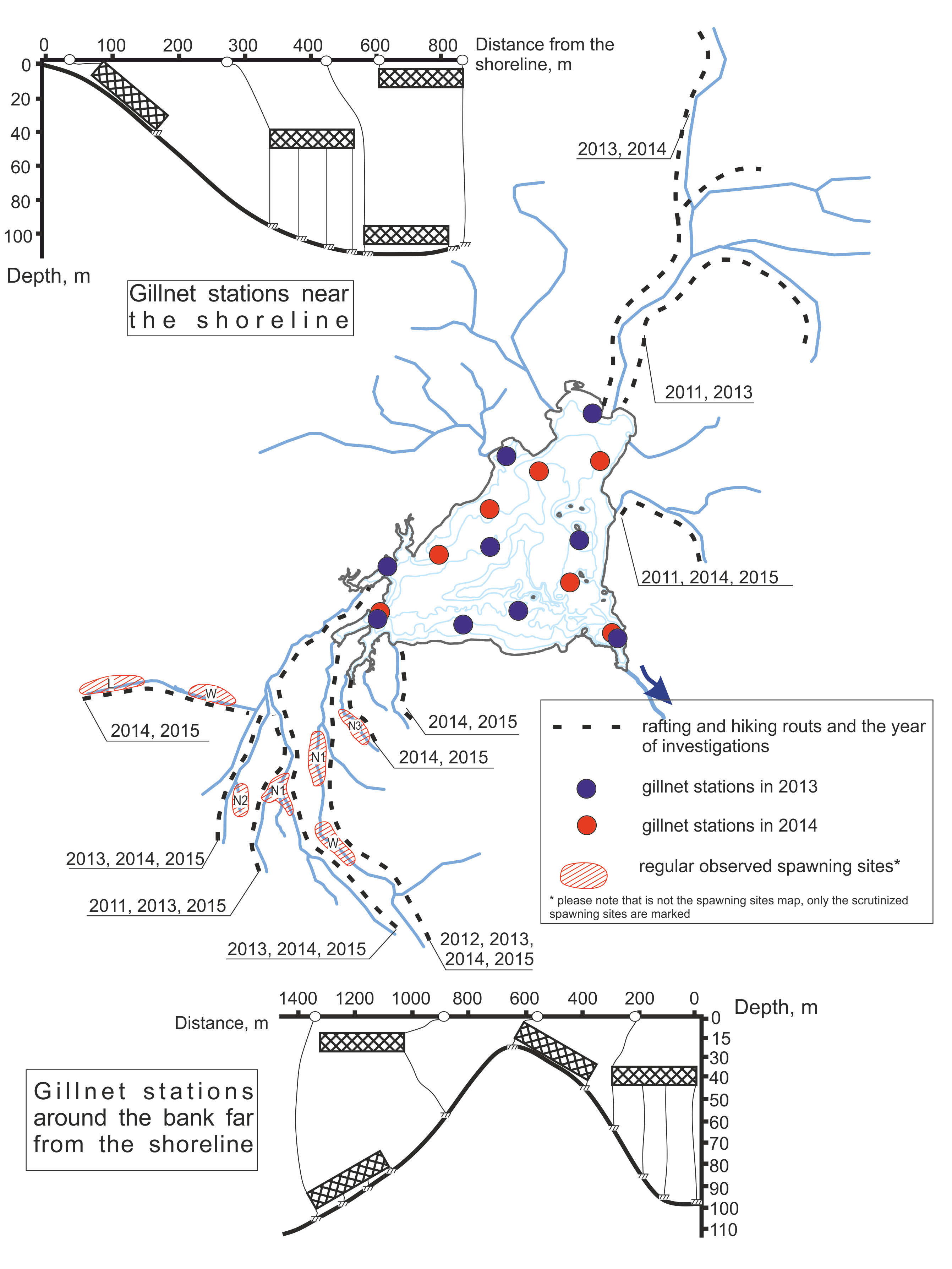


**Figure S2.** Locations of 23 + 10 anatomical landmarks used to capture shape for geometric morphometric analyses


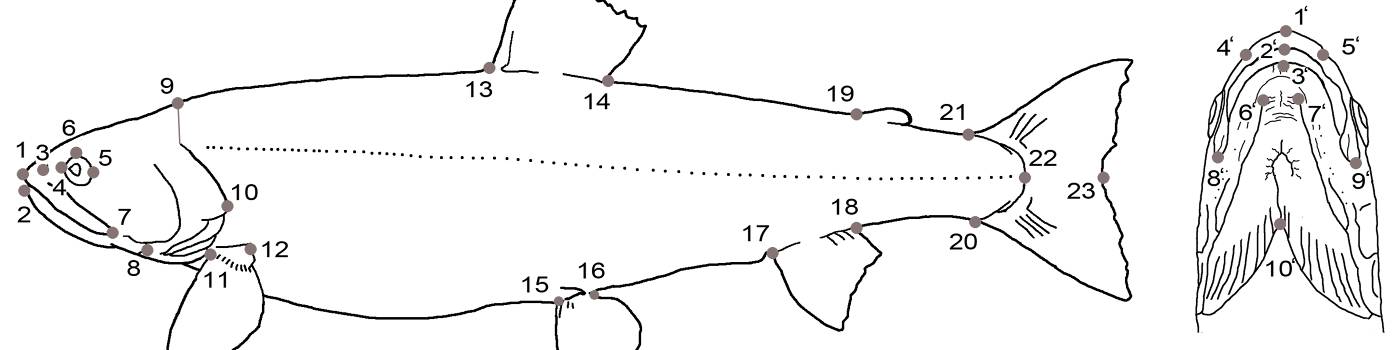


**Table S1.** Jack-knife classification matrix of morphs – comparison of *a priori* classification by head proportions and classification after multivariate analysis of side-view shape through CVA. Values in bold indicate the number of individuals correctly classified to the appropriate morph

| CVA classification | *A priori* classification | | | | | | | | |
| --- | --- | --- | --- | --- | --- | --- | --- | --- | --- |
|  | W | L | N | < N1 | N2 | N3 > | S | B | Dolly Varden |
| W | **48** | 4 | 11 | - | - | - | 0 | 0 | 9 |
| L | 2 | **31** | 0 | - | - | - | 0 | 0 | 0 |
| N | 6 | 0 | **126** | - | - | - | 2 | 0 | 3 |
| < N1 | - | - | - | **63** | 1 | 1 | - | - | - |
| N2 | - | - | - | 2 | **40** | 1 | - | - | - |
| N3 > | - | - | - | 2 | 1 | **29** | - | - | - |
| S | 1 | 0 | 2 | - | - | - | **41** | 0 | 1 |
| B | 0 | 0 | 0 | - | - | 0 | 0 | **47** | 0 |
| % Correct overall: | 84 | 89 | 91 | 94 | 95 | 94 | 95 | 100 | 52 |

**Table S2**. Head morphometry of Lake Kronotskoe charrs and anadromous Dolly Varden (% of head length: M ± SD / Lim)

| Traits | Morphs (spec.) | | | | | | | |
| --- | --- | --- | --- | --- | --- | --- | --- | --- |
|  | W  (57) | L  (35) | N1  (67) | N2  (42) | N3  (31) | S  (43) | B  (47) | Dolly Varden  (27) |
| Snout length  (1–4) | 18.5 ± 0.22  15.4–22.4 | 24.4 ± 0.58  18.0–31.9 | 18.0 ± 0.26  14.0–22.9 | 22.2 ± 0.35  18.3–27.3 | 25.0 ± 0.48  20.0–29.4 | 15.7 ± 0.29  11.7–20.3 | 17.0 ± 0.31  12.9–22.4 | 22.0 ± 0.33  17.3–25.9 |
| Eye diameter  (4–5) | 23.7 ± 0.27  20.2–28.4 | 21.9 ± 0.57  18.2–27.1 | 24.2 ± 0.23  20.5–29.6 | 23.5 ± 0.23  20.8–27.3 | 20.0 ± 0.36  16.0–24.4 | 31.5 ± 0.30  28.7–35.6 | 26.8 ± 0.32  21.9–31.0 | 21.9 ± 0.48  19.0–25.7 |
| Operculum length  (5–10) | 56.6 ± 0.41  52.7–62.2 | 60.8 ± 0.42  54.0–66.8 | 59.3 ± 0.32  53.6–66.2 | 58.9 ± 0.29  53.6–62.5 | 54.4 ± 0.66  44.5–59.4 | 53.8 ± 0.33  49.5–56.5 | 57.2 ± 0.30  53.8–61.8 | 57.8 ± 0.44  52.8–63.3 |
| Upper jaw length  (1–7) | 54.1 ± 0.52  45.7–62.7 | 57.2 ± 0.73  47.6–67.1 | 43.0 ± 0.39  35.4–51.9 | 50.4 ± 0.47  46.0–56.2 | 51.0 ± 0.55  44.9–58.1 | 44.5 ± 0.43  39.7–50.5 | 49.9 ± 0.51  43.8–57.6 | 53.7 ± 0.55  44.1–60.3 |
| Lower jaw length  (2–8) | 66.5 ± 0.53  59.1–78.9 | 67.5–0.66  59.4–76.9 | 58.4 ± 0.50  51.1–67.0 | 52.0 ± 0.50  46.1–58.3 | 48.5 ± 1.08  35.1–56.4 | 62.7 ± 0.63  53.9–73.2 | 69.9 ± 0.50 62.2–79.1 | 64.9 ±0.63  56.6–77.8 |
| Max. head height  (8–9) | 70.8 ± 0.67  57.8–82.7 | 63.6 ± 0.78  56.2–76.5 | 79.0 ± 0.54  70.8–87.5 | 75.1 ± 0.62  67.3–87.8 | 73.1 ± 0.84  67.8–83.3 | 73.3 ± 0.75  65.1–83.6 | 70.5 ± 0.71  48.5–79.4 | 70.7 ± 0.75  56.3–83.7 |
| Rostrum length  (1’–2’) | - | - | 8.7 ± 0.34  4.2–11.9 | 11.5 ± 0.51  5.6–18.5 | 9.7 ± 0.53  5.4–16.2 | - | - | - |
| Width between nostrils (4’–5’) | - | - | 31.3 ± 0.49  25.6–38.4 | 27.8 ± 0.56  21.1–34.3 | 28.4 ± 0.60  22.1–35.6 | - | - | - |
| Width between tips of the upper jaw (8’–9’) | - | - | 52.0 ± 1.01  38.0–66.3 | 53.7 ± 1.33  41.0–70.8 | 44.2 ± 1.09  33.8–55.0 | - | - | - |
| Front angular length  (7’–9’) | - | - | 33.3 ± 0.54  25.8–42.8 | 37.5 ± 0.91  22.4–47.7 | 32.5 ± 0.61  27.4–39.5 | - | - | - |
| Back angular length  (9’–10’) | - | - | 35.8 ± 0.43  29.1–44.4 | 32.3 ± 0.43  26.7–36.9 | 31.3 ± 0.46  27.1–35.9 | - | - | - |

**Table S3.** CVA outputs for morphological shape variation

| Root | Comparison of five main  morphs (L, W, N, S, B) | | | Comparison of three N-morphs | | | | | |
| --- | --- | --- | --- | --- | --- | --- | --- | --- | --- |
|  |  |  |  | side view | | | head ventral view | | |
|  | Eigenvalues | % Variance | Cumulative % | Eigenvalues | % Variance | Cumulative % | Eigenvalues | % Variance | Cumulative % |
| 1 | 6.981 | 41.291 | 41.291 | 6.287 | 72.204 | 72.204 | 1.867 | 52.107 | 52.107 |
| 2 | 4.914 | 29.067 | 70.358 | 1.829 | 22.533 | 94.737 | 1.479 | 42.203 | 94.310 |
| 3 | 2.011 | 11.893 | 82.251 | 0.873 | 5.263 | 100 | 0.395 | 5.690 | 100 |
| 4 | 1.180 | 6.977 | 89.228 | - | - | - | - | - | - |
| 5 | 0.875 | 5.173 | 94.401 | - | - | - | - | - | - |
| 6 | 0.579 | 3.427 | 97.828 | - | - | - | - | - | - |
| 7 | 0.367 | 2.172 | 100 | - | - | - | - |  | - |

**Table S4.** Canonical loadings of landmarks on main CV roots

| Landmarks | Comparison of five  main morphs  (L, W, N, S, B) | | | Comparison of three N-morphs | | | |
| --- | --- | --- | --- | --- | --- | --- | --- |
|  |  |  |  | side view | | head ventral view | |
|  | CV1 | CV2 | CV3 | CV1 | CV2 | CV1 | CV2 |
| 1 | 0.0473 | 0.0616 | 0.2227 | 0.2641 | 0.0105 | 0.0445 | 0.0090 |
| 2 | 0.3018 | 0.1614 | 0.0600 | 0.6263 | 0.1458 | 0.0693 | 0.0111 |
| 3 | 0.0542 | 0.1185 | 0.1738 | 0.1216 | 0.2745 | 0.0356 | 0.0152 |
| 4 | 0.1608 | 0.1165 | 0.2064 | 0.3684 | 0.4243 | 0.0292 | 0.0213 |
| 5 | 0.1244 | 0.0859 | 0.1578 | 0.4222 | 0.4355 | 0.0215 | 0.0182 |
| 6 | 0.0421 | 0.1301 | 0.1234 | 0.2841 | 0.1377 | 0.0291 | 0.0239 |
| 7 | 0.0676 | 0.1412 | 0.1213 | 0.1418 | 0.2455 | 0.0248 | 0.0358 |
| 8 | 0.3415 | 0.0207 | 0.0250 | 0.4563 | 0.1546 | 0.0212 | 0.0583 |
| 9 | 0.0934 | 0.0544 | 0.0755 | 0.0721 | 0.0650 | 0.0074 | 0.0598 |
| 10 | 0.0295 | 0.0963 | 0.0912 | 0.0804 | 0.1230 | 0.0229 | 0.0164 |
| 11 | 0.0424 | 0.0852 | 0.0903 | 0.2132 | 0.1076 |  |  |
| 12 | 0.0478 | 0.0993 | 0.0321 | 0.1744 | 0.0379 |  |  |
| 13 | 0.0640 | 0.0065 | 0.0316 | 0.1295 | 0.1345 |  |  |
| 14 | 0.0198 | 0.0375 | 0.1465 | 0.1579 | 0.1043 |  |  |
| 15 | 0.0442 | 0.0576 | 0.0763 | 0.1509 | 0.1306 |  |  |
| 16 | 0.0291 | 0.0687 | 0.1164 | 0.1381 | 0.0841 |  |  |
| 17 | 0.0398 | 0.0586 | 0.0264 | 0.1158 | 0.0995 |  |  |
| 18 | 0.0452 | 0.0257 | 0.0555 | 0.6105 | 0.1789 |  |  |
| 19 | 0.0581 | 0.0535 | 0.0959 | 0.5726 | 0.1815 |  |  |
| 20 | 0.1538 | 0.0843 | 0.0457 | 0.1356 | 0.5591 |  |  |
| 21 | 0.1088 | 0.0263 | 0.1145 | 0.1853 | 0.4956 |  |  |
| 22 | 0.1421 | 0.0424 | 0.0540 | 0.1074 | 0.1137 |  |  |
| 23 | 0.0224 | 0.1011 | 0.0499 | 0.0709 | 0.0865 |  |  |

**Table S5**. Lake Kronotskoe morphs occurrence (%) and maximal catch (ind. h^-1^ 10m^-2^) in the different ecosystem zones

| Morph | Litoral and sublitoral (0–20 m) | | Epilimnion (0–20 m) | | Hypolimnion (>20 m) | | Profundal (>40 m) | |
| --- | --- | --- | --- | --- | --- | --- | --- | --- |
|  | % | $\frac{ind.}{10\times m^{2}\times h}$ | % | $\frac{ind.}{10\times m^{2}\times h}$ | % | $\frac{ind.}{10\times m^{2}\times h}$ | % | $\frac{ind.}{10\times m^{2}\times h}$ |
| W | 38 | 42.4 × 10^-2^ | 75 | 33.1 × 10^-2^ | 30 | 11.1 × 10^-2^ | 32 | 23.5 × 10^-2^ |
| L | 1 | 1.2 × 10^-2^ | 14 | 6.3 × 10^-2^ | 2 | 0.8 × 10^-2^ | 0 | 0 |
| B | 4 | 4.6 × 10^-2^ | 2 | 0.9 × 10^-2^ | 16 | 5.6 × 10^-2^ | 58 | 43.3 × 10^-2^ |
| N1 | 38 | 43.1 × 10^-2^ | 6 | 2.5 × 10^-2^ | 2 | 0.7 × 10^-2^ | 5 | 3.5 × 10^-2^ |
| N2 | 13 | 14.6 × 10^-2^ | 1 | 0.3 × 10^-2^ | 0 | 0 | 0 | 0 |
| N3 | 6 | 6.8 × 10^-2^ | 1 | 0.4 × 10^-2^ | 0 | 0 | 0 | 0 |
| S | 0 | 0 | 1 | 0.3 × 10^-2^ | 50 | 18.6 × 10^-2^ | 5 | 3.9 × 10^-2^ |
